# Directed Evolution of Proteins through *In Vitro* Protein Synthesis in Liposomes

**DOI:** 10.1155/2012/923214

**Published:** 2012-08-16

**Authors:** Takehiro Nishikawa, Takeshi Sunami, Tomoaki Matsuura, Tetsuya Yomo

**Affiliations:** ^1^ERATO Japan Science and Technology (JST) and Yomo Dynamical Micro-Scale Reaction Environment Project, Graduate School of Information Sciences and Technology, Osaka University, 1-5 Yamadaoka, Suita, Osaka 565-0871, Japan; ^2^Graduate School of Information Science and Technology, Osaka University, 1-5 Yamadaoka, Suita, Osaka 565-0871, Japan; ^3^Graduate School of Engineering, Osaka University, 2-1 Yamadaoka, Suita, Osaka 565-0871, Japan; ^4^Graduate School of Frontier Biosciences, Osaka University, 1-5 Yamadaoka, Suita, Osaka 565-0871, Japan

## Abstract

Directed evolution of proteins is a technique used to modify protein functions through “Darwinian selection.” *In vitro* compartmentalization (IVC) is an *in vitro* gene screening system for directed evolution of proteins. IVC establishes the link between genetic information (genotype) and the protein translated from the information (phenotype), which is essential for all directed evolution methods, by encapsulating both in a nonliving microcompartment. Herein, we introduce a new liposome-based IVC system consisting of a liposome, the protein synthesis using recombinant elements (PURE) system and a fluorescence-activated cell sorter (FACS) used as a microcompartment, *in vitro* protein synthesis system, and high-throughput screen, respectively. Liposome-based IVC is characterized by *in vitro* protein synthesis from a single copy of a gene in a cell-sized unilamellar liposome and quantitative functional evaluation of the synthesized proteins. Examples of liposome-based IVC for screening proteins such as GFP and **β**-glucuronidase are described. We discuss the future directions for this method and its applications.

## 1. Introduction

Protein engineering is a technology that tailors a protein to function in a desired way. Rational design and directed evolution are two major approaches for introducing a change into the amino acid sequence of proteins. As a small change in the protein sequence can induce critical functional changes in proteins, altering the amino acid sequence is a crucial step in these approaches; the amino acid sequences are primarily altered by introducing mutations in the gene that encodes the protein of interest. In site-directed mutagenesis, specific mutations to the DNA sequence are introduced, which yields a desired function if the relationship between protein structure and function is clearly understood. However, directed evolution of proteins is based on Darwinian selection and thus does not necessarily require knowledge of the relationship between protein sequence and function [1, 2]. Using this method, mutations are generated through techniques such as random mutagenesis, recombination, or site-directed diversification [3]. Subsequently, the protein variants are synthesized from the mutated genes using living hosts (cells) or an *in vitro* transcription-translation system (IVTT), and they are screened for the desired function. Therefore, the methods used for directed evolution can be categorized as “*in vivo*” and “*in vitro*” approaches. 

**Figure 1:**
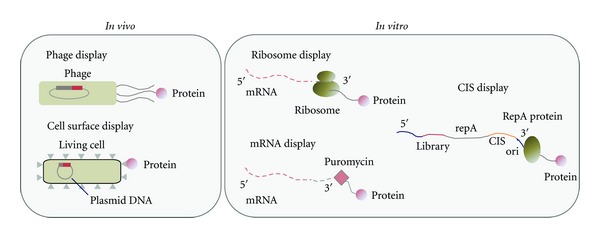
Genotype (genetic information)-phenotype (protein synthesized from the gene and its function) linkage and screening techniques for directed evolution of proteins. Screening techniques are categorized as *in vivo* and *in vitro* approaches.

The difference between these two approaches (*in vivo* and *in vitro* approach) is the way that the genotype (genetic information encoding a protein) and a phenotype (the protein synthesized from the gene and its function) are linked for the genes of interest (Figure 1). Through an *in vivo* approach, the genotype-phenotype link is produced by using a living cell. For example, cell-surface display is an *in vivo* screening technique that uses a fusion protein to localize protein molecules to a cell membrane surface. Target proteins fused with a membrane protein are displayed on the cell membrane surface, screened for the desired function by exposure to a colorimetric detection reagent, and selectively sorted using a fluorescence-activated cell sorter (FACS) [4, 5]. Phage display is another *in vivo* display technique that uses a phage for gene storage and protein display. In this technique, target proteins are fused with phage coat proteins (*g8p* or *g3p*) and displayed on a phage surface. These *in vivo* screening techniques have been applied to the directed evolution of proteins. However, these techniques are applicable to a limited number of proteins that are not toxic to growth of the host cell. Low transformation efficiency also limits genetic diversity (library size) by up to 10^8^. 

**Figure 2:**
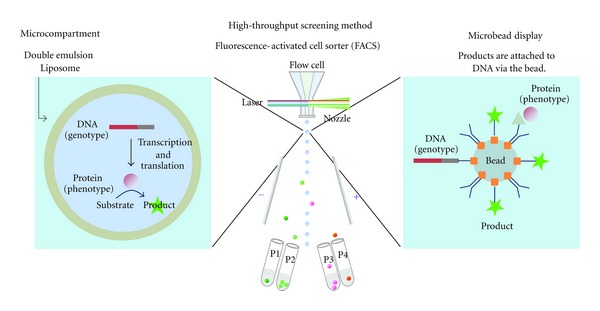
The underlying concept for *in vitro* compartmentalization (IVC) using double emulsion or liposome (left) and microbead (right). In both cases, a fluorescence-activated cell sorter (FACS) (center) is used for high throughput screening.

To overcome these technical drawbacks in *in vivo* techniques, *in vitro* display was proposed as a new display technique [6, 7]. In this technique, protein variants are synthesized from the gene using an IVTT, and the gene (genotype) is physically or covalently tethered to the translated protein (phenotype) via an adaptor or linker, such as ribosomes (ribosome display) [8], RepA (CIS display) [9], and puromycin (mRNA display) [10]. The proteins linked to the mutant gene are screened for the desired function. These *in vitro* display methods are suitable for improving protein equilibrium affinity, off rate, stability, and folding [8]. However, these display techniques are not suitable for improving the catalytic activity of enzymes because they rely on binding affinity between the displayed protein and an immobilized ligand for the screen [11]. *In vitro* compartmentalization (IVC) is a solution to direct screening for enzymatic reaction turnover entirely *in vitro*. The primary idea underlying IVC is that a DNA, an IVTT, and a fluorogenic detection reagent are encapsulated in a cell-like compartment to form a genotype-phenotype linkage (Figure 2, left). Proteins are translated from a single gene using an IVTT in each compartment, and they yield a fluorescent product that is screened directly for the catalytic activity of interest using an FACS [11]. We introduce herein the earlier studies on IVC-based directed evolution of proteins, where water-in-oil (W/O) emulsions were used as microcompartments. We then introduce the IVC using cell-sized lipid vesicles, liposomes. Firstly, the technology underlying protein synthesis using an IVTT inside liposome is described. Then, construction of the liposome-based gene screening system using FACS and examples of the application of the liposome-based IVC to directed evolution of proteins are described. Finally, we remark on the future directions for liposome-based IVC in directed evolution that are impossible with other IVC techniques.

## 2. *In Vitro* Compartmentalization (IVC)

### 2.1. Emulsion-Based IVC

*In vitro* compartmentalization (IVC) is a technique for linking genotype to phenotype. Unlike other techniques used in conventional *in vitro* display, IVC does not connect directly the gene and encoded protein. IVC utilizes microcompartments for genotype-phenotype linkage. A single DNA and an IVTT are encapsulated in a microcompartment (Figure 2, left). Proteins encoded by the gene accumulate inside the microcompartment through *in vitro* protein synthesis. Colocalization of the gene and protein links the genotype and phenotype. W/O emulsion was first utilized for microcompartments in IVC-based genetic screening. With this technique, genes encoding the DNA methyltransferase *M. HaeIII* were enriched from a mixture containing 10^7^-fold excess genes encoding dihydrofolate reductase [12]. Furthermore, toward high-throughput gene screening using an FACS, microbead display using IVC (Figure 2, right) was performed to screen catalytic activity of enzymes with a soluble non-DNA substrate [13]. This technique enables us to evaluate the catalytic activity of enzyme encapsulated in cell-size microcompartments under a variety of conditions that can inhibit the *in vitro* protein synthesis, because the evaluation of catalytic activity is separated from the protein synthesis. As a next advancement of IVC, water-in-oil-in-water emulsion (double emulsion) was adapted and enabled direct sorting of intact emulsion droplets. This double emulsion technique was first demonstrated through model selection of emulsion droplets encapsulating *FolA* genes from a droplet mixture with two separate W/O emulsions: a positive emulsion containing *FolA* genes and a fluorescent marker as well as a negative emulsion containing *M. HaeIII* genes and no fluorescent marker [14]. Reemulsification of W/O emulsion droplets in the aqueous phase creates double emulsion droplets, which can be directly analyzed and sorted using an FACS. Using the emulsion-based IVC and *in vitro* protein synthesis, Ebg, which is an *E. coli* protein of unknown function, was evolved into mutant proteins with *β*-galactosidase catalytic activity [15]. Single genes from the mutation library for *Ebg* as well as an IVTT and a fluorogenic substrate were compartmentalized in a W/O emulsion droplet. In an emulsion droplet, Ebg variants are translated from the mutant gene and yield fluorescent product if the variants express *β*-galactosidase catalytic activity. After reemulsification of the W/O emulsion in the aqueous phase, double emulsion droplets were screened directly for *β*-galactosidase activity (through the fluorescent signal from turnover reaction products). 

### 2.2. Advantages and Limitations of Emulsion-Based IVC

**Figure 3:**
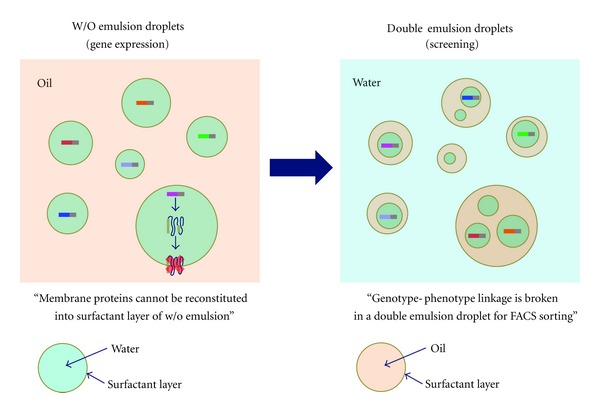
The technical limitations of emulsion-based IVC due to the “W/O emulsion” and “double emulsion” structures.

Emulsion-based IVC is suitable for the quantitative screening of enzyme variants using an FACS because each emulsion droplet yields a fluorescent signal, which reflects the enzymatic activity of each variant. Other *in vitro* display techniques involve screening based on a binding event between a displayed protein and immobilized ligand and are not adapted for observing a catalytic turnover event. Although emulsion-based IVC has been useful and successful for directed evolution of enzymes, this method has two technical limitations. The first limitation concerns the stringency of the genotype-phenotype link (Figure 3, right). Double emulsion droplets containing multiple compartments are formed when W/O emulsion is reemulsified in an aqueous phase. During the reemulsification process, two types of microcompartments can be entrapped in a double emulsion droplet; one microcompartment can encapsulate the gene of interest and the other can encapsulate an unrelated gene. The genotype-phenotype link would be severed if two different mutant genes were in a double emulsion droplet for sorting using an FACS [16]. One approach to overcome the issues from multiple compartments is through a high-throughput screening platform using droplet-based microfluidics [16]. This screening platform comprises a droplet generation device (droplets for gene amplification), droplet fusion device (electrocoalescence between droplet pairs of a gene-containing droplet and an IVTT-containing droplet for the genotype-phenotype link), and sorting device (for recovery of the genes of interest). The second limitation of the emulsion-based IVC is a technical hurdle for its application using a variety of protein classes, such as membrane proteins (Figure 3, left), which cannot be overcome by the use of the aforementioned droplet-based microfluidics. 

For the technical issue of single droplet containing substructures, and that preclude membrane protein use in directed evolution of proteins (Figure 3), cell-sized microcompartments with a phospholipid bilayer membrane are ideal solution for both issues [17–19]. We have been studying *in vitro* protein synthesis in liposomes [20–23] and constructed a high-throughput gene screening system using liposomes (liposome-based IVC) and an FACS [24, 25]. Our experimental system for protein synthesis in liposomes comprises a liposome as the bioreactor, chemical components for protein synthesis, and analytical tools for quantitation of the proteins produced. The following sections survey the liposomes used in preparation methods for cell-sized compartments (Section 3), *in vitro* protein synthesis using a PURE system (Section 4), high-throughput analysis using an FACS (Section 5), and liposome-based IVC for directed evolution of proteins (Section 6).

## 3. Liposomes as Cell-Sized Microcompartments

### 3.1. Liposomes

**Figure 4:**
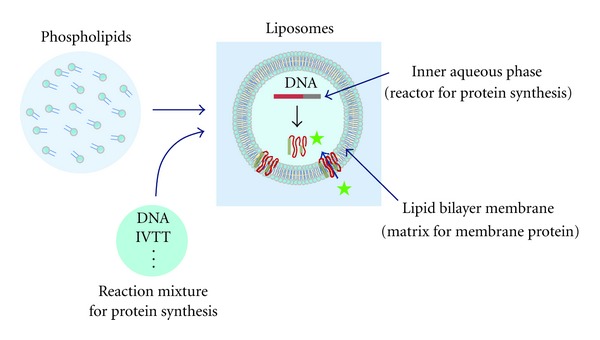
Liposomes as a platform for *in vitro* protein synthesis. Reaction mixture for protein synthesis constitutes an inner aqueous phase of liposome, in which protein is synthesized from a DNA. Membrane protein synthesized inside liposome can be embedded into a lipid bilayer membrane.

A phospholipid vesicle is a spherical hollow capsule that has an inner aqueous phase surrounded by a phospholipid bilayer membrane. The vesicular structure is formed spontaneously by dispersing phospholipids in an aqueous medium (Figure 4). Vesicle formation from egg lecithin was first reported by Bangham and Horne in 1964 [26]. They observed dried samples from an aqueous dispersion of lecithin by electron microscopy and discovered a spherical structure with a 4.4 nm thick lipid layer of lamellae. “Liposome” is a term for a phospholipid vesicle and was proposed by Sessa and Weissmann in 1968 [27]. This term is generally accepted. Since the first report by Bangham, liposomes have been utilized in various biophysical and biochemical studies, including model membranes, microreactors, supramolecular assemblies for biomimetic systems, and drug carriers for drug delivery systems [28]. Currently, liposome-related studies are motivated by a growing interest in “synthetic cells” [29] and the “origin of life” [29], both of which are intended to address how living things might emerge from nonliving matter [30]. The recent trend in liposome-related studies regards liposomes as a protocell model in which biochemical reactions inside a living cell are executed by filling liposomes with the required components. *In vitro* protein synthesis in liposomes and its application to genetic screening (liposome-based IVC) are examples of bioengineering as well as liposome-related studies. 

### 3.2. Preparation Methods for Cell-Sized Liposomes

Liposomes are diverse in size (from several tens of nm to hundreds of *μ*m in diameter), lamellarity (singly lamellar or multilamellar), and internal structure (single compartment or multiple compartments). This diverse structure depends on the liposome preparation methods. However, not all sizes of liposomes are applicable as microcompartments for IVC-based gene screening due to detection limits (approximately 1 *μ*m in diameter) in FACS measurements (see Section 5 for details). Therefore, the liposome size suitable for this experiment is similar to a cell size (larger than 1 *μ*m in diameter), and such a cell-sized liposome is referred to as a “giant liposome” [31]. Giant liposomes are primarily generated using the “hydration of thin film method,” “rehydration of freeze-dried empty liposome (FDEL) method,” or “inverted-emulsion method” [32].

In liposome-based IVC, a single DNA and an IVTT are encapsulated in the same giant liposome to link genotype and phenotype (Figure 4). Feasibility of liposome preparation, encapsulation of the reactants for *in vitro* protein synthesis, and the internal structure of the liposome are significantly influenced by the preparation method for the giant liposome. For the hydration of thin film or rehydration of freeze-dried empty liposome methods of giant liposome preparation, the liposomes are formed by reconstituting dried lipid film with a reaction buffer for protein synthesis. The advantage of the hydration of thin film method is that liposomes can be prepared using various phospholipids irrespective of electrical charge. However, the disadvantage of the method is that a relatively large quantity of reaction mixture is necessary for swelling the dried thin film of the lipids during liposome preparation, and macromolecular compounds in the reaction mixture are difficult to trap in the liposomes [32]. When giant liposomes are prepared by the rehydration of freeze-dried empty liposomes, the liposome structure is sufficiently strong to withstand the osmotic pressure change in the outer solution and the sorting operation during the FACS screen [33]. However, the giant liposomes generated by this method are unsuitable for quantitative evaluation of in-liposome protein synthesis using an FACS because certain giant liposomes have multiple compartments and lamella; thus, the liposome size measured using an FACS does not represent the compartment size for protein synthesis [34]. Consequently, liposomes with a single compartment and lamella (e.g., giant unilamellar liposomes) are required for quantitative evaluation of in-liposome protein synthesis (liposome size and product quantity) by high-throughput analysis using an FACS. 

The inverted-emulsion method is a preparation method for giant unilamellar liposomes [35]. This method comprises the following steps. The aqueous phase is emulsified in an oil phase containing phospholipids to prepare a water-in-oil emulsion. The emulsion is layered on an outer aqueous solution and centrifuged to sediment the emulsion droplets towards the oil-water interface where a lipid monolayer forms. The emulsion droplets generate a second lipid layer upon crossing the interface and transform into unilamellar liposomes in the outer aqueous solution. When this type of giant unilamellar liposome is applied to *in vitro* protein synthesis, the compartmentalized reaction mixture is separated from the outer aqueous phase until the liposomes are formed. Thus, researchers can control the composition of both inner and outer aqueous phases. The inverted-emulsion method is promising for construction of a suitable microcompartment to quantitatively evaluate in-liposome protein synthesis and reconstitution of membrane proteins.

## 4. Protein Synthesis in Liposomes

### 4.1. The PURE System for *In Vitro* Protein Synthesis

The primary components for *in vitro* protein synthesis are DNA encoding the protein of interest, an IVTT, and a detection reagent. These components should be encapsulated firmly in a liposome when *in vitro* protein synthesis is performed in liposomes (Figure 4). An IVTT is a multimolecular machine that facilitates protein synthesis from DNA in a test tube. Cell extracts from *E. coli*, wheat germ, rabbit reticulocytes, and insect cells have been used as IVTTs for *in vitro* protein synthesis [36]. However, cell extracts comprise certain unknown constituents. Furthermore, proteases, DNase, RNase, and intrinsic enzymes (e.g., *β*-galactosidase) remain in the cell extracts, and these remnants considerably decrease the production of protein and interfere with the detection of protein function. These problems are inevitable as long as cell extracts are used for *in vitro* protein synthesis. To overcome these problems, we have been using the IVTT developed by reassembling the individual components for protein synthesis, which were extracted from *E. coli* cells overexpressing the protein factors with a histidine tag and thoroughly purified. This new IVTT is referred to as a “protein synthesis using recombinant elements (PURE) system” [37]. *In vitro* protein synthesis is a coupled reaction system comprising transcription, aminoacylation of tRNA, translation, and energy source regeneration. The PURE system includes the entire reaction system and is prepared by reconstituting protein factors, ribosomes, tRNA mixture, and substrates (20 amino acids and four nucleoside triphosphates) in a buffer solution. The protein factors are T7 RNA polymerase, pyrophosphatase, 20 aminoacyl-tRNA synthetases, creatine kinase, myokinase, and nucleoside diphosphate kinase in addition to 10 translation factors (three initiation factors (IF), three elongation factors (EF), three release factors (RF), and a ribosome recycling factor (RRF)). 

### 4.2. Protein Synthesis from a Single Gene in a Liposome

Using the experimental system with liposomes, DNA, and an IVTT, *in vitro* protein synthesis in liposomes has been studied by a number of groups [31]. The review article by Stano et al. [31] is a comprehensive survey of biomacromolecule synthesis in liposomes for the creation of semisynthetic minimal cells and provides the most recent and comprehensive list of publications on protein synthesis inside liposomes. Thus, our primary focus is on protein synthesis that begins with a single gene in a liposome, which is a crucial part of liposome-based IVC, because genotype and phenotype must be linked for the gene screening process. Our strategy, which links genotype and phenotype inside a liposome, includes DNA that encodes the protein of interest and is encapsulated at a single molecule level with the PURE system in liposomes. Liposomes in which green fluorescent protein (GFP) was translated from a single gene were successfully detected, analyzed, and sorted for a fluorescence signal from GFP using an FACS [24]. *β*-glucuronidase catalytic activity expressed from a single gene inside the liposomes was also detected and quantitatively evaluated using a fluorogenic substrate and FACS [34]. 

## 5. High-Throughput Analysis of Liposomes Using an FACS

### 5.1. Application of an FACS to Liposome Measurement

In the liposome-based IVC, an extremely large number (more than 10^8^) of liposomes are created for *in vitro* protein synthesis beginning with a single DNA. Liposomes that encapsulate a gene of interest are screened from the large population of liposomes for the desired protein function. The protein function expressed inside an individual liposome should be detected and quantitatively analyzed to identify the liposomes for sorting. Protein production and function inside the liposome are often measured and quantified using analytical tools such as a fluorescence spectrometer and fluorescence microscope [31]. A fluorescence spectrometer detects an averaged fluorescence signal from an ensemble of liposomes. A fluorescence microscope measures a fluorescence signal from an individual liposome where proteins are synthesized from genes. Microscopy measurements for liposomes provide data on the morphology (shape and size) and fluorescence intensity of a liposome (internal reaction). However, this technique is only effective for a much smaller liposome population than the population required for statistical analysis and gene screening in IVC-based directed evolution of proteins. FACS is a promising technique for observing a large population of liposomes because of its capacity for high-throughput analysis and simultaneous measurement of multiple characteristics. 

An FACS is a powerful experimental apparatus for analyzing and sorting live cells simultaneously. The apparatus comprises a fluidics system for transporting one cell at a time, an interrogation system for detecting the cell by laser illumination, and a sorting system for collecting the cells of interest from one to millions of cells. Using this technique, cells exhibiting a specific biological characteristic are separated from a heterogeneous population of cells using fluorescence and light scattering from individual cells in the population. The FACS was invented in the late 1960s, commercialized in the early 1970s, and has been utilized since then for basic studies in cell biology as well as clinical applications such as diagnosis, disease classification, and *in vivo* therapies [38]. Recently, FACS measurements have not only been used for cell-oriented applications but also for molecular screening in directed evolution of proteins [15, 39]. In addition, the FACS has been utilized for measuring nonbiological particles such as submicron-size liposomes [40] and double emulsion droplets [41] for particle size and fluorescent marker entrapment. We have used FACS to characterize liposomes for structure and biochemical reactions. 

**Figure 5:**
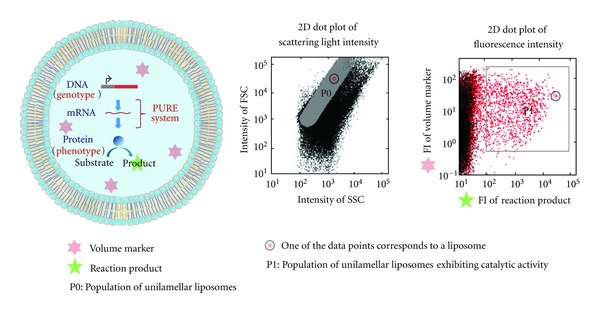
Characterization of in-liposome protein synthesis using an FACS. DNA, PURE system, fluorogenic substrate, and fluorescent volume marker are encapsulated in a giant unilamellar liposome (left). Subpopulation of unilamellar liposomes is represented by P0 in 2D dot plot of scattering light intensity (middle). Catalytic activity of enzymes expressed in liposomes is confirmed in P1 region of 2D dot plot of fluorescence intensity obtained by FACS measurement (right).

We first successfully detected a GFP synthesis in liposomes using FACS based on fluorescence signals from the synthesized GFP [22]. For liposome structure, the internal aqueous volume and membrane volume of individual liposomes were quantitatively evaluated using light scattering intensity data from an FACS measurement [42]. The liposome population selected using these structural parameters was sorted using an FACS and observed by optical microscopy. The structural parameters generated using the FACS correlated with liposome structural heterogeneity, as demonstrated by microscopy observations. Population analysis of giant liposomes with an FACS was used to identify the subpopulation of unilamellar liposomes in a 2D contour map of surface area and internal aqueous volume generated for giant liposomes [43] (Figure 5). Furthermore, substructure of the multilamellar giant liposomes has been identified by encapsulating *β*-glucuronidase synthesis in liposome, and analyzed by an FACS [34]. 

### 5.2. Evaluation of an In-Liposome Reaction Using FACS

Analysis of biochemical reactions in liposomes using an FACS is based on a quantitative evaluation of liposome size and reaction products in liposomes. Liposome size is evaluated by measuring the fluorescence intensity of a fluorescent protein as a volume marker molecule, which is encapsulated in a liposome at a high concentration [24, 34]. The fluorescence intensity of the marker protein is converted to the number of marker molecules in a liposome and then to volume of the internal aqueous phase in the liposome. The reaction product is quantified by measuring the fluorescence intensity of new synthesized proteins or the fluorescent product of expression of a protein function [20, 25]. Through this analytical method using an FACS, a large population of liposomes can be measured for size and reaction as well as analyzed throughout a population or subpopulation that is defined by reactivity and a specific size [33]. For liposome-based IVC enzyme screening, a fluorescent volume marker and fluorogenic substrate are encapsulated in liposomes for a screening assay using an FACS (Figure 5, left). Details on this screening system are described in Section 6. 

Using our system, GFP synthesis inside the liposomes was quantitatively evaluated, and the influence of lipid membrane composition on protein synthesis was discussed [44]. The study suggested that phospholipids and other liposomal membrane components for liposome preparation should neither inhibit nor impair the protein synthesis reaction steps. Furthermore, GFP synthesis inside the liposomes proceeds similarly to that in the test tube in spite that liposomes have very high surface-to-volume ratio in comparison to a test tube. This indicates that phospholipids and other liposomal membrane components for liposome preparation neither inhibit nor impair the protein synthesis reaction steps [20]. Consequently, liposome provides a reaction environment that is equally good as a test tube and provides an extremely large number (more than 10^10^/100 *μ*L reaction volume) of microcompartments.

## 6. Liposome-Based IVC for Directed Evolution of Proteins

### 6.1. Liposome-Based IVC

We constructed a novel gene screening system using a liposome-based IVC for directed evolution of proteins [24, 25]. Liposome-based IVC is a technique used to link genotype and phenotype. The idea underlying this technique is that a mutant gene library is compartmentalized as a single molecule into cell-sized liposomes (giant liposomes) and a protein variant is synthesized from the encapsulated mutant gene through the PURE system in each liposome. Liposome-based IVC has two primary advantages over emulsion-based IVC. Unlike W/O emulsion droplets, liposomes are directly loaded onto an FACS apparatus when they are analyzed for gene screening (no reemulsification process is required for FACS analysis). The catalytic activity of an enzyme expressed in a liposome is quantitatively evaluated using an FACS when a giant unilamellar liposome is utilized for enzyme synthesis and catalytic activity expression. An FACS measurement collects signals from two or more different fluorescence colors (a fluorescent volume marker and fluorogenic substrate) from individual liposomes simultaneously and quantitates the liposome size and reaction product concentration, both of which are necessary for quantitative evaluation of catalytic activity. In addition, membrane proteins can be inserted into the phospholipid bilayer membrane of a liposome when giant unilamellar liposome is utilized for membrane protein synthesis. Membrane protein incorporated into lipid bilayer membrane is a prerequisite for quantitative evaluation of membrane protein function and subsequent genetic screening. 

We first performed a pilot experiment for liposome-based IVC and demonstrated that the technique is promising for genetic screens [24]. Two GFP variants, GFPuv2 and GFPuv5, were used in the pilot experiment, and they were encoded in the pETG2tag and pETG5tag vectors, respectively. GFPuv5 emits a fluorescent signal eight times higher than GFPuv2 when excited at 488 nm. A mixture of the pETG2tag and pETG5tag DNA at molar ratio of 0.85 : 0.15 was compartmentalized into giant liposomes with the PURE system and a fluorescent volume marker. Giant liposomes were prepared by FDEL method (Section 3). After incubation for GFP synthesis, the liposomes were measured for fluorescent signals from the translated GFP as well as volume marker and sorted using the higher fluorescent intensity of GFP and a certain liposome size. The pETG5tag was enriched over 10-fold from the initial genetic mixture when the liposomes were collected from two liposome subpopulations; one subpopulation ranged from 1.4fL to 6.7fL and the other ranged from 6.7fL to 13fL. Therefore, the genotype- (GFP gene-) phenotype (GFP) link was securely constructed in individual liposomes, which encapsulated a single copy of DNA. Thus, the pilot experiment successfully showed that GFP genes encapsulated in a liposome can be screened for the fluorescence intensity from GFP emission. 

**Figure 6:**
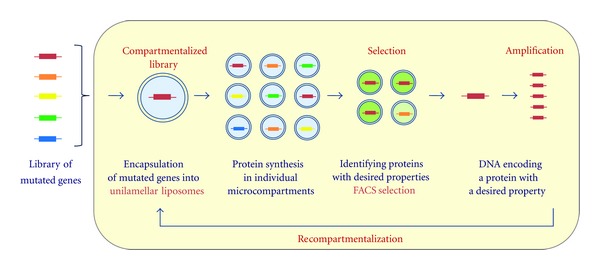
Flow chart for protein screening using liposome-based IVC. Library of mutated genes are compartmentalized with PURE system and other reagents into liposome. Each protein variant is synthesized from a single copy of gene in each liposome. Liposomes encapsulating the gene of interest are screened using an FACS. DNA extracted from the liposomes is amplified to be transferred to the next round of gene screening.

However, we anticipated the following technical issue, which can be caused by multiple compartments and lamella in giant liposomes prepared by the FDEL method [34]. The issue is underestimation of catalytic activity where a gene is expressed only in a subset of the multiple compartments in a giant liposome. This yields an inaccurate evaluation of catalytic activity in individual liposomes and lower enrichment in the gene of interest. In addition, translocation of a membrane protein into the membrane of a multilamellar liposome is a technical hurdle for detection of a functional membrane protein. To solve these problems, we used a giant unilamellar liposome for liposome-based IVC. A giant unilamellar liposome was prepared using the inverted-emulsion method (Section 3.2). We constructed a genetic screening system composed of *in vitro* protein synthesis encapsulated within a giant unilamellar liposome and an FACS (Figure 6). A mock genetic library for *β*-glucuronidase (GUS) was compartmentalized into liposomes as a single molecule. The liposomes that exhibited green fluorescence from hydrolysis of the fluorogenic substrate through the synthesized GUS were sorted from the subpopulation of giant unilamellar liposomes using an FACS. More than a 10-fold enrichment of the GUS gene with a higher catalytic activity was generated when a single copy of the GUS gene was encapsulated in each liposome. Quantitative analysis of the enrichment factors and their liposome size dependencies showed that the experimentally generated and theoretical values agreed. Using this method, the genes encoding active GUS were then enriched from a gene library of randomly mutated GUS genes. Only three rounds of screening were required, which was also consistent with our theoretical estimation. The consistency between the theoretical and experimental values generated using our screening system indicates that the screening system operates as expected. 

### 6.2. Protein Evolution Directed by Compartment Size

Here, the directed evolution of protein is discussed through the effect of compartment size on protein function. Nature contains living prokaryotes with cell sizes that range from 0.02fL to 400fL [45]. The lower limit of the cell size is determined by the catalytic efficiency of enzymes, protein synthesis machinery, and machinery to cope with sudden environmental changes [45]. Under this theory, smaller cells could be generated if the enzyme catalytic efficiency was greater. Naturally occurring proteins have evolved in the cell through Darwinian selection. However, directed evolution of protein has never been discussed regarding compartment size because conventional microcompartments (emulsions) have been unsuitable for this purpose. Of the gene screening techniques for directed evolution of proteins, liposome-based IVC is the most promising technique for studying how compartment size influences protein evolution because the internal aqueous phase volume of the liposome is accurately evaluated by FACS measurement. A molecular evolution system using liposome-based IVC is an experimental approach for simulating the evolutionary process of protein function in a certain cell size. 

We propose a molecular evolution system for evaluating the effect of compartment size on protein evolution. The system comprises a giant unilamellar liposome, GUS, and an FACS. Giant unilamellar liposomes are polydisperse in size ranging from 0.5fL to 250fL or larger, which includes the cell size discussed. GUS is a tetrameric enzyme, and GUS tetramer formation is a rate limiting step in catalytic activity expression [46]. Kinetic analysis of GUS tetramer formation in emulsion droplets showed that tetramer formation is susceptible to compartment size when GUS is synthesized from a single gene in a W/O emulsion droplet [47]. Monomeric GUS is prone to assemble in a smaller compartment because tetramer formation is the rate limiting step. In our molecular evolution system, a library of randomly mutated GUS genes and the PURE system are compartmentalized in giant unilamellar liposomes. GUS variants are synthesized in individual liposomes. Liposomes exhibiting GUS catalytic activity are sorted from the subpopulation defined by a certain liposome size (100fL) and green fluorescence intensity above threshold value. We predict that GUS variants prone to assemble in a larger compartment (100fL) will be generated after iterative rounds of genetic screening. Our genetic screening experiment is in progress and will continue until it generates a gene encoding active GUS variants, which are fit to a certain liposome size. 

### 6.3. Adaptation of Membrane Protein Function to a Liposome Environment via Directed Evolution

Membrane proteins perform a variety of functions in cells, including material transport, signal transduction, and cell-cell contact. With recent progress in minimal cell research using liposomes and an IVTT, experimental methods for including membrane proteins are under development. Giant unilamellar liposomes are an ideal cell-mimetic environment because the lipid composition can be optimized for reconstitution of membrane proteins. Although certain water soluble proteins have been synthesized using an IVTT inside liposomes (Section 4), thus far only a few membrane proteins have been synthesized inside liposomes and reconstituted into a lipid bilayer membrane. For synthesis of membrane proteins inside liposomes, a few groups have succeeded in synthesizing and characterizing *α*-hemolysin for membrane permeation of nutrient molecules in giant unilamellar liposomes [18] as well as sn-glycerol-3-phosphate acyltransferase (GPAT) and lysophosphatidic acid acyltransferase (LPAAT) for lipid synthesis in liposomes [48]. We believe that through liposome-based IVC development, *in vitro* molecular evolution of membrane proteins become possible. Advantages of using liposome-based IVC on the molecular evolution of membrane proteins are expected as follows. (1) Various kinds of membrane proteins can be engineered irrespective of their toxicity threatening cells' lives, (2) functions of membrane proteins can be evaluated under various reaction conditions and also in membranes with various lipid compositions. Thus, research on membrane proteins is entering a new stage for applications aimed at complex molecular machines, such as biosensors for a monitoring device, biochips for diagnosis, and biointerfaces for computing.

## 7. Conclusions

In this paper, we reviewed a novel *in vitro* genetic screening system comprising liposome-based IVC and an FACS. Liposome-based IVC is a new technique developed to link genotype and phenotype. This technique utilizes a giant unilamellar liposome and a PURE system. A library of mutant genes and a PURE system are compartmentalized into giant unilamellar liposomes for *in vitro* protein synthesis. A protein variant (phenotype) translated from a single DNA is colocalized with the DNA (genotype) inside a liposome. Using an FACS for high-throughput screening, liposomes encapsulating the gene of interest are sorted from a large population of liposomes using fluorescent signals generated from expression of a protein function. The genes of interest are enriched through iterative rounds of genetic screening. 

With the gene screening system, genetic diversity at approximately 10^7^ can be screened in a day [25]. The diversity size is sufficiently large for directed evolution of proteins. The system can screen various proteins including enzymes and membrane proteins. A large population of liposomes of different sizes (from 0.5fL to 250fL) facilitates the search for a protein function that has adapted to a certain compartment size. In addition, the semipermeable character of the liposomal membrane facilitates external feeding of a liposome compartment using additional solutes. If the protein function of interest is coupled to the external solute, then the protein screen is controlled by the timing of feeding and/or solute quantity. 

Liposome-based IVC was successfully proven effective for screening a protein function under simple conditions. However, in nature, proteins must have evolved to adapt to more complex and dynamic environments where many biochemical reactions are coupled and organized to control cell behavior. This suggests that a reaction system comprising many proteins and enzymes can evolve to perform more efficiently and more productively. Liposome-based IVC will be a useful method for simulating versatile conditions by assembling the necessary components into a liposome reactor. It is expected that such liposome reactors containing a coupled reaction system have high potential as biochemical sensors for monitoring chemicals (e.g., carcinogens, toxins, and environmental hormones) and microreactors to produce biologically active substances for daily use (e.g., anticancer drugs and antibiotics) with high efficiency and selectivity.
